# Supplementary material for: Whole-of-school physical activity implementation in the context of the Dubai Fitness Challenge
Source: PLoS One. 2024 Mar 13;19(3):e0290856. doi: 10.1371/journal.pone.0290856 (PMC10936810; doi:10.1371/journal.pone.0290856)
Supplement: Attachment — Submitted filename: PLos ONE Journal Review report.docx [file pone.0290856.s005.docx]

# PLOS ONE Journal Reviewer Report

**Title:** Whole-of-School Physical activity Implementation in the Context of the Dubai Fitness Challenge

**Manuscript ID**: PONE-D-23-25904

**Type of Manuscript**: Research article

**1. Introduction**

The report is composed of different aspects that are necessary for decision-making regarding the readiness of the manuscript to be considered for publication by the PLOS ONE Journal. It outlines the introduction, summary of the research and my overall impression, the strong points, discussion of specific areas for improvement (weaknesses, major issues and minor issues), other points that need to be considered, and recommendations.

**2**. **Summary of the research and my overall impression**

Even though the manuscript has some weaknesses that will be outlined later, it has strong points. Consequently, my overall impression of the manuscript is that it is publishable in the PLOS ONE Journal. The manuscript is based on the study that was conducted focusing on the Whole-of- school Physical activity in Dubai.

**3**. **Strong points of the manuscript**

The strong points are that:

1. The manuscript is in line with the aim, the scope and publication criteria of the Journal. The number of words on the abstract is within the acceptable range. It focuses on an area that is very important for different stakeholders, which include students, teachers and the community regarding physical activity.
2. There is consistency in terms of the way in which sources have been referenced.
3. Good attempt in terms of the way in which the manuscript has been edited.
4. The knowledge gap has been clearly identified in page 7, line 171 and line 172.
5. Ethical issues on informed consent and consent from parents/guardians have been included as outlined in line 224 and line 225.
6. There is a clear indication of the way in which each of the research questions have been answered.
7. There is a detailed explanation of the way in which data were generated and analysed to answer the research questions.
8. Quotations have been used to give voice to the participants
9. Sources have been used under discussion to either support or negate the argument
10. The authors have outlined the limitations of the study.
11. The conclusion is based on the data, results and the discussion section of the study. However, it can be improved by including other aspects in line with the research questions and themes that were generated. The proposal for the improvement has been outlined under weaknesses.

**4.**     **Discussion of specific areas for improvement (weaknesses, major issues and minor issues)**

**Weaknesses**

Strong points notwithstanding, the manuscript has weaknesses that need to be addressed to improve its quality and acceptability for publication by the PLOS ONE Journal. Weaknesses are that:

**4.1 Major Issues**

1. The study used the Whole-of-School Physical Activity approach as a lens; however, there is no indication of the way in which the approach guided the authors with regard to the selection of the research design, sampling and research methods.

2. The authors have selected a convergent parallel mixed-methods research design; however, there is no justification for the selection of this kind of a design.

3. In page 10, line 222, the authors have blocked the name of the ethics board that gave ethical clearance for the study, and that breaks the flow of the sentence. I suggest that the authors use a pseudonym to represent the name of the institution than to block it.

4. In line 236 and 237, the authors indicate that the students were able to complete the survey during normal classroom time with the assistance of teachers. The question is “Did the assistance by teachers not compromise the data?” Which steps were taken to ensure that data generation is not compromised? Did the assistance by teachers during normal teaching time not disrupt teaching and learning by teachers and students?

5. What were the disadvantages of using volunteer participants as reflected in page 13, line 279?

**4.2 Minor issues**

There may be a need to include parental engagement and the usefulness of using a whole-of-school PA lens on a study in the conclusion section.

**5. Other points**

The authors have also omitted other ethical aspects that are necessary for the study of this nature. For example, privacy and confidentiality, anonymity, benefits, risks and harm, and assent from the students.

**6.**     **Recommendations**

My recommendation is minor revisions
